# Interrelationship Between Obstructive Sleep Apnea Syndrome and Small Airway Disease: A Comprehensive Review

**DOI:** 10.3390/biomedicines13040905

**Published:** 2025-04-08

**Authors:** Chou-Chin Lan, Chung Lee, Lun-Yu Jao, Yao-Kuang Wu, Kuo-Liang Huang, Wen-Lin Su, Yi-Chih Huang, Chih-Wei Wu, Mei-Chen Yang

**Affiliations:** 1 Division of Pulmonary Medicine, Taipei Tzu Chi Hospital, Buddhist Tzu Chi Medical Foundation, New Taipei City 23142, Taiwan; 2 School of Medicine, Tzu Chi University, Hualien 97004, Taiwan

**Keywords:** obstructive sleep apnea syndrome, small airway disease, comorbidities, personalized treatment strategies

## Abstract

**Study Objectives:** This review aims to explore the epidemiology, pathophysiology, risk factors, and diagnostic approaches of obstructive sleep apnea syndrome and small airway disease, emphasizing their interrelationship and implications for clinical management. **Methods:** A comprehensive analysis of the literature was conducted to examine shared and distinct characteristics of obstructive sleep apnea syndrome and small airway disease. Risk factors, clinical presentations, diagnostic tools, and management strategies were reviewed to identify potential areas for improvement in care. **Results:** Obstructive sleep apnea syndrome, characterized by intermittent upper airway obstruction during sleep, contributes to fragmented sleep and systemic diseases. Small airway disease involves inflammation and obstruction of the small airways, impairing airflow and gas exchange. Shared risk factors, such as obesity, smoking, and age, were identified as contributors to the development and progression of both conditions. The co-occurrence of obstructive sleep apnea syndrome and small airway disease exacerbates respiratory symptoms and increases the risk of comorbidities, such as pulmonary hypertension, heart failure, and respiratory failure. Recognition of their interplay highlights the need for integrated diagnostic and therapeutic strategies. **Conclusions:** The interrelationship between obstructive sleep apnea syndrome and small airway disease underscores the importance of integrated management approaches to improve patient outcomes. Addressing shared risk factors and understanding the interplay between these conditions are crucial for optimizing care. This review identifies key knowledge gaps, including the need for precise diagnostic tools and targeted therapies, which are essential for advancing personalized treatment strategies for individuals with obstructive sleep apnea syndrome and small airway disease.

## 1. Introduction

### 1.1. Overview of Obstructive Sleep Apnea Syndrome (OSAS)

OSAS is a common sleep disorder characterized by recurrent episodes of complete or partial obstruction of the upper airway during sleep, leading to breathing pauses, reduced airflow, and frequent awakenings [1]. These apneic events often result in a significant decrease in blood oxygen levels, causing fragmented sleep and daytime fatigue. OSAS is associated with a range of risk factors, including obesity, anatomical abnormalities of the airway, and age, and can lead to serious health consequences, such as cardiovascular disease, metabolic syndrome, and impaired cognitive function. This condition not only affects the quality of life but also poses substantial risks for various comorbidities, making its recognition and management critical for overall health [1].

### 1.2. Overview of Small Airway Disease (SAD)

In contrast to OSAS, which is primarily an upper airway disorder, SAD encompasses a spectrum of conditions involving small airways, defined as those with a diameter < 2 mm [2]. SAD is characterized by inflammation, obstruction, and airway remodeling. These small airways are crucial for proper lung function as they facilitate airflow and gas exchange. SAD is often associated with chronic respiratory conditions such as asthma and chronic obstructive pulmonary disease (COPD) [2]. The symptoms of SAD include coughing, wheezing, and shortness of breath, particularly during exertion [2]. The pathophysiology of SAD involves a combination of inflammatory processes, structural changes, and increased airway resistance, which can lead to impaired lung function and respiratory distress [2]. The early detection and management of small airway diseases are essential to prevent progression and improve patient outcomes, particularly in individuals with comorbid conditions such as OSAS, where overlapping mechanisms may exacerbate respiratory symptoms and overall health.

### 1.3. Importance of Studying the Relationship Between OSAS and SAD

Studying the association between OSAS and SAD is crucial for several reasons. First, both conditions significantly affect respiratory function and overall health, often coexisting and exacerbating each other’s symptoms [1,2]. Understanding this interplay can lead to better diagnostic approaches and more effective treatment strategies tailored to the specific needs of individuals. Second, the presence of SAD in individuals with OSAS may contribute to increased airway resistance, worsening the severity of apneic events, and leading to heightened cardiovascular risk and metabolic dysfunction [3]. By exploring this relationship, researchers can identify potential biomarkers and therapeutic targets that simultaneously address both conditions simultaneously [4,5]. Additionally, a deeper understanding of how OSAS influences the small airways can inform public health initiatives aimed at improving respiratory health, particularly in at-risk populations. Ultimately, advancing knowledge in this area may enhance patient outcomes, reduce healthcare costs, and promote a holistic approach for managing sleep-related and respiratory disorders. Although numerous studies have explored the overlap between OSAS and conditions, such as COPD or asthma [6,7], SAD is distinct from these disorders. Therefore, it is critical to understand the relationship between OSAS and SAD.

While few previous studies have addressed the association between OSAS and SAD, this review offers a more comprehensive analysis and comparison, providing a novel perspective that further enhances the understanding of these conditions.

## 2. Epidemiology

### 2.1. OSAS Prevalence

The prevalence of OSAS varies across populations owing to factors such as age, sex, ethnicity, and body mass index. In the general adult population, the prevalence of OSAS ranges from 9% to 38% for an apnea–hypopnea index (AHI) of ≥5/h and from 6% to 17% for an AHI of ≥15/h [8]. The prevalence of OSAS is notably higher among obese individuals (body mass index [BMI] ≥ 30.9 kg/m^2^ for men and BMI > 31.7 kg/m^2^ for women) that approximately 69% have any degree of OSA (AHI ≥ 5/h), while 32% have moderate-to-severe OSAS (AHI ≥ 15/h) [9]. Sex differences were also prominent, as male participants were more likely to develop OSAS (AHI ≥ 5/h) than female participants, with a male-to-female ratio of 3.5:1 [10]. However, its prevalence in female participants increases after menopause, which narrows the sex gap [11]. The prevalence of OSAS is higher in individuals with chronic respiratory diseases [12,13]. In individuals with asthma, the prevalence of OSAS, defined by an AHI > 5/h, was approximately 68% [12]. OSAS, defined by an AHI > 5/h, is present in 50% of individuals with COPD, with its prevalence increasing as disease severity progresses [13].

### 2.2. SAD Prevalence

The overall prevalence of SAD in the general population remains unclear as it varies significantly depending on population characteristics, underlying medical conditions, and diagnostic criteria [14,15]. The lack of standardized definitions and diagnostic tools contributes to challenges in obtaining precise statistics, and SAD is often underdiagnosed and underreported. Epidemiological studies have shown that small airway dysfunction is often identified in individuals with respiratory distress or chronic cough, underscoring the importance of awareness and early assessment. In a study conducted in China, spirometry-defined small airway dysfunction was highly prevalent with an overall rate of 43.5% [14]. The prevalence of small airway obstruction varies widely, ranging from 5% in Tartu, Estonia, to 34% in Mysore, India [15]. Overall, although the precise prevalence rates for SAD alone are difficult to establish, its significant association with respiratory diseases highlights its relevance in clinical practice and public health.

The prevalence of SAD is associated with risk factors such as smoking, environmental pollutants, and occupational exposures [14]. In smokers, SAD can develop early, even before the onset of significant airflow obstruction, which is typically diagnosed as COPD [14]. Small airway involvement is present in over 50% of individuals with COPD, which is a common condition associated with SAD [16]. The prevalence of SAD increases with disease severity and is linked to impaired lung function and a higher amount of symptoms [16]. Similarly, in asthma, a substantial proportion of individuals may exhibit small airway dysfunction, with estimates indicating that approximately 40% to 70% of individuals with asthma have significant small airway disease, particularly those with more severe or persistent symptoms [17].

### 2.3. Prevalence of SAD Among OSAS Individuals

The prevalence of SAD and OSAS overlap remains unclear and is an emerging area of research. However, previous studies have indicated a significant overlap between these two conditions. In individuals with OSAS, SAD is often associated with increased respiratory symptoms and reduced lung function [18]. In OSAS, small airway function is impaired, likely due to breathing at low lung volumes and the cyclic closure and reopening of small airways [18]. This dysfunction may influence the natural progression. Obesity, a key risk factor for both OSAS and SAD, further exacerbates small airway involvement in these individuals [19]. In obese OSAS individuals, small airway resistance is elevated, as indicated by impulse oscillometry (IOS) values [19]. The co-occurrence of SAD and OSAS underscores the need for a comprehensive understanding of their shared pathophysiologies.

## 3. Pathophysiology

### 3.1. Pathophysiology of OSAS

The pathophysiology underlying OSAS involves a complex interplay of anatomical, physiological, and environmental factors that lead to intermittent upper airway obstruction during sleep [20]. Anatomical predispositions such as an enlarged uvula, increased neck circumference, or retrognathia can narrow the airway, making it more susceptible to collapse during sleep, particularly when muscle tone is reduced [20]. This reduction in muscle tone, especially during rapid eye movement (REM) sleep, allows airway collapse to be exacerbated by chronic inflammation that can lead to airway edema and increased resistance [21]. Additionally, the negative intrathoracic pressure created during apneic events places a greater workload on the respiratory muscles, further promoting airway obstruction [20]. Obesity significantly contributes to OSAS because excess fat around the neck can narrow the airways and activate inflammatory pathways [9]. The sleep position also plays a role, with the supine position being particularly detrimental owing to the gravitational effects on the soft tissues of the throat [22]. Finally, comorbid conditions, such as asthma and COPD, can complicate the clinical presentation of OSAS [6,7]. This highlights the need for a comprehensive understanding of these mechanisms to improve diagnosis and treatment strategies.

### 3.2. Pathophysiology of SAD

The mechanisms of SAD involve various pathological processes that lead to inflammation, obstruction, and remodeling of the small airways, which are essential for effective pulmonary function [23]. Chronic inflammation is a hallmark of SAD and is often triggered by exposure to irritants such as cigarette smoke, environmental pollutants, or allergens [23]. Inflammation recruits immune cells that release cytokines and mediators, thereby promoting tissue damage and airway constriction. Prolonged inflammation can lead to structural changes, including airway remodeling, fibrosis, increased smooth muscle mass, and mucus hypersecretion, which collectively narrow the airways and increase resistance [23]. This leads to a significantly limited airflow and impaired gas exchange. Additionally, goblet cell hyperplasia results in excessive mucus production, which obstructs the small airways, further promoting inflammation and infection. Bronchoconstriction, in which the smooth muscles surrounding the airways contract, can also occur in response to various stimuli, leading to increased airway resistance [23]. As the small airways become obstructed and remodeled, gas exchange efficiency decreases, resulting in hypoxemia and hypercapnia, which manifest as shortness of breath and exercise intolerance [23]. Understanding these mechanisms is crucial for the effective diagnosis and management of SAD, especially in individuals with overlapping conditions such as OSAS, where the interplay between these diseases complicates treatment and outcomes.

### 3.3. Possible Pathophysiology Linking OSAS and SAD

The pathophysiology linking OSAS and SAD involves a complex interplay of anatomical and pathological factors that contribute to airway obstruction. In OSAS, hypoxia–reoxygenation leads to systemic hypoxemia and oxidative stress, which damage cellular structures such as lipids, proteins, and DNA, perpetuating inflammation and promoting airway remodeling and obstruction—key features of SAD [24,25]. Inflammatory mediators released during this process further contribute to airway inflammation, mucus hypersecretion, and bronchoconstriction [26]. Oxidative stress and inflammation impair epithelial function, disrupting mucociliary clearance and exacerbating mucus accumulation, leading to symptoms such as coughing, wheezing, and dyspnea [24,25,26]. The increased negative intrathoracic pressure generated during apneic episodes can cause dynamic airway collapse, affecting both large and small airways [27], while also promoting thoracic fluid accumulation, further narrowing the small airways and increasing respiratory resistance. Obesity, a common risk factor for both OSAS and SAD, exacerbates airway narrowing due to fat deposition around both large and small airways, increasing the likelihood of obstruction [9,19].

Overall, the interplay between recurrent hypoxia–reoxygenation, oxidative stress, inflammation, and mechanical factors such as negative intrathoracic pressure and fluid shifts contributes to airway remodeling, impaired mucociliary function, and progressive respiratory dysfunction in OSAS and SAD.

## 4. Risk Factors

### 4.1. Risk Factors Associated with the Development of OSAS

Several risk factors contribute to the development of OSAS [28]. The most common risk factors include obesity and excessive fat deposition around the neck and upper airways, which can lead to airway narrowing and increased resistance during sleep [28]. Other significant risk factors include age, with a higher prevalence in middle-aged and older adults, and sex, as men are more likely to develop OSAS than women [28]. Family history and genetic predisposition also play a role, as individuals with a family history of OSAS are at increased risk. Additional factors, such as smoking, alcohol consumption, nasal congestion, and anatomical abnormalities (e.g., enlarged tonsils and deviated septum) can further predispose individuals to OSAS [28].

### 4.2. Risk Factors Associated with the Development of SAD

SAD is influenced by a range of risk factors, including chronic exposure to environmental pollutants, such as cigarette smoke, air pollution, and occupational hazards [15]. These factors contribute to inflammation and damage to small airways. Obesity is another important risk factor, because excess body fat can contribute to increased airway resistance and remodeling [15]. Genetic predispositions may also play a role in the development of SAD, particularly in individuals with a family history of respiratory conditions. Other conditions such as asthma, COPD, and gastroesophageal reflux disease (GERD) have been linked to an increased risk of SAD due to their inflammatory and obstructive effects on the airways [15].

### 4.3. Risk Factors Associated with the Development of SAD in OSAS

Several interconnected risk factors influence the development of SAD in individuals with OSAS. These factors exacerbate airway inflammation and obstruction, leading to impaired respiratory function. The key risk factors include the following:

1. Obesity: Obesity, a shared risk factor for both OSAS and SAD, contributes to fat deposition around the airways, increasing airway resistance and exacerbating airway inflammation [9,19].

2. Age: Older age was associated with a higher prevalence of OSAS and SAD. Aging can lead to structural changes in both the upper and lower airways as well as a decline in respiratory muscle strength, which increases the risk of airway obstruction [28,29]. Small airway disease increases with age, increasing by approximately 2% per decade after the age of 50 years, even among never-smokers with normal lung function and no respiratory symptoms [29].

3. Comorbid respiratory diseases: Individuals with asthma or COPD are at an increased risk of developing both SAD and OSAS. The prevalence of OSAS is known to be higher in asthma and COPD [12,13]. In individuals with COPD, approximately 50% of those with SAD, with the prevalence of SAD increasing with COPD severity, reported as 14.3%, 51.1%, 91%, and 100% across GOLD grades 1 to 4, respectively [30]. The prevalence of SAD ranged from 53% to 64% in individuals with mild-to-moderate asthma and from 75% to 90% in those with severe asthma [31]. Given the high prevalence of both OSAS and SAD in asthma and COPD, there is a significant coexistence of OSAS and SAD in these individuals.

## 5. Evaluating Approaches for OSAS and SAD

### 5.1. Tools for Evaluating OSAS

The evaluation of OSAS involves a combination of clinical evaluations, questionnaires, and diagnostic tests. The following methods are commonly used to evaluate OSAS.

1. Clinical Assessment: A thorough medical history is essential, including details of the patient’s sleep patterns, snoring, witnessed apneas, excessive daytime sleepiness, and any associated symptoms, such as fatigue or cognitive impairment [32]. Clinicians may also assess risk factors such as obesity, age, and comorbid conditions [32].
2. Sleep Questionnaires: Common sleep questionnaires, such as the Epworth Sleepiness Scale or Pittsburgh Sleep Quality Index, can help evaluate the severity of daytime sleepiness and overall sleep quality and provide valuable insights into the likelihood of OSAS [32]. The STOP-Bang questionnaire is a widely used screening tool for OSAS [33]. It assesses key risk factors including snoring, daytime tiredness, observed apnea, high blood pressure, body mass index, age, neck circumference, and sex. The STOP-Bang’s straightforward scoring system helps identify individuals at low, intermediate, or high risk for OSAS, making it a valuable complement to the ESS and PSQI in evaluating individuals with suspected sleep apnea [33].
3. Polysomnography (PSG): PSG is the gold standard for evaluating OSAS and is typically performed overnight in sleep laboratory [34]. During PSG, various physiological parameters are recorded, including electroencephalography to monitor brain activity and identify sleep stages; electromyography to measure muscle activity, particularly in the chin and legs; and electrooculography to track eye movements for sleep stage assessment. Respiratory effort is monitored by observing chest and abdominal movements, whereas oxygen saturation is measured using pulse oximetry to assess blood oxygen levels [34]. Additionally, airflow through the nose and mouth is evaluated using a nasal cannula or thermal sensor to assess breathing patterns. Heart rate and electrocardiography were monitored during PSG to assess cardiovascular function during sleep. The AHI derived from PSG was used to quantify the severity of OSAS, based on the number of apneas and hypopneas per hour of sleep. The AHI categories were classified as mild (5–15 events per hour), moderate (15–30 events per hour), or severe (>30 events/h). This index helps clinicians assess the degree of respiratory disturbance during sleep and guides treatment decisions in individuals with OSAS [34].
4. Home Sleep Apnea Testing (HSAT): HSAT is an alternative evaluating tool for OSAS that allows individuals to undergo sleep monitoring in the comfort of their homes [34]. HSAT typically involves the use of portable devices that record key physiological parameters, such as respiratory effort, airflow, oxygen saturation, and heart rate during sleep. Although it is more convenient and less expensive than in-laboratory PSG, HSAT is generally recommended for individuals with a high pre-test probability of OSAS and no significant comorbidities. However, HSAT may have limitations in evaluating other sleep disorders or in individuals with complex medical conditions because it typically provides fewer data points than PSG. Despite these limitations, HSAT is a useful tool for evaluating OSAS in many individuals [34].
5. Sleep Endoscopy: A minimally invasive procedure in which a flexible tube with an endoscope is inserted into the nasal passages to visualize the nasal cavity and oropharynx for anatomical abnormalities or obstructions [35]. Sleep endoscopy, such as drug-induced sleep endoscopy, involves sedating the patient and using an endoscope to visualize the airway collapse during sleep. It helps assess which parts of the airway are obstructed and can guide surgical planning if necessary [35].
6. Cephalometric Radiography: A specialized radiograph of the head is used to analyze the skeletal structure, including the relationships between the jaw, airway, and soft tissue, which can contribute to sleep apnea [36].
7. Modified Mallampati Classification: Evaluating maxillofacial and nasopharyngeal structures, including the modified Mallampati classification, is beneficial for assessing OSAS [37]. By assessing factors such as tongue size, palatal abnormalities (e.g., enlarged tonsils or uvula), and mandibular or maxillary anomalies, clinicians can better understand the severity of airway obstruction. This classification provides valuable insight into potential anatomical obstructions in the upper airway that may contribute to OSAS [37].
8. Maxillofacial or Neck Computed Tomography (CT): To assess OSAS, a maxillofacial or neck CT scan is commonly used [36]. These scans provide detailed imaging of the facial bones, soft tissues, and airway structures, allowing for the evaluation of bony anomalies, soft tissue hypertrophy (e.g., enlarged tonsils and adenoids), and the cross-sectional area of the airway to identify potential anatomical obstructions contributing to OSAS [36].
9. Magnetic Resonance Imaging (MRI): MRI offers high-resolution imaging of soft tissues, making it valuable for evaluating the soft palate, tongue, pharyngeal walls, and other structures that contribute to airway obstruction in OSAS [36]. Additionally, MRI can assess the brain structures involved in sleep regulation and provide insights into potential central apnea components. Its ability to visualize both anatomical and neurological factors makes it a useful tool for the comprehensive assessment of sleep-disordered breathing [36].
10. Ultrasound: Ultrasound is an emerging tool for evaluating airway structures in OSAS that offers a non-invasive and radiation-free approach [38]. It is particularly useful for assessing the size, position, and mobility of the tongue base as well as for visualizing soft tissues such as the soft palate, uvula, and lateral pharyngeal walls, which may contribute to airway obstruction [38]. Additionally, the submandibular and hyoid bone regions should be examined to evaluate their roles in airway patency. Dynamic imaging of the anterior neck allows the observation of airway changes during breathing or phonation, providing real-time insights into potential obstructions. By replicating sleep conditions through supine or lateral positioning, ultrasound enables the practical assessment of anatomical and functional contributors to OSAS, offering a complementary option to established imaging modalities [38].

### 5.2. Tools for Evaluating SAD

1. Clinical Assessment: A comprehensive evaluation of individuals with respiratory conditions begins by obtaining a detailed history and conducting a thorough physical examination [39]. History focuses on symptoms such as cough, wheezing, and dyspnea while also identifying potential risk factors such as smoking, occupational exposure, and family history of respiratory diseases. Physical examination often includes auscultation of the lungs, which may reveal abnormal sounds, such as wheezing or decreased breath sounds, providing important clues about the underlying condition and its severity [39].
2. Pulmonary Function Test (PFT) Spirometry: A widely used tool for evaluating lung disease severity [39]. Obstructive lung disease was evaluated when the FEV1/FVC ratio was <70%, although FEV1 can be influenced by factors such as lung volume, elastic recoil, and effort. While FEV1 primarily reflects large airway obstruction, significant small airway disease must accumulate before abnormalities appear [39]. FEF25–75, which measures the mid-portion of expiratory flow, is often used to detect small airway pathologies. This reflects the resistance of the smaller airways during the later stages of exhalation. However, FEF25–75 has limitations, including dependence on FVC, poor reproducibility, and sensitivity issues [39]. The forced expiratory volume in 3 s (FEV3) to the FVC ratio (FEV3/FVC ratio) and the fraction of air not expired in the first 3 s (1-FEV3/FVC) are alternative measures with better accuracy in detecting SAD, especially in older individuals [40]. These measures assess the proportion of air not expired in the first 3 s, offering improved sensitivity compared to FEF25–75 [40].
3. Plethysmography: Plethysmography provides sensitive measures of air trapping and lung hyperinflation, which are often observed in obstructive lung disease [40]. Hyperinflation, which is characterized by an elevated lung volume at the end of expiration, results from airflow limitation, reduced lung elastic recoil, and altered chest wall compliance. Prolonged expiration and airway closure lead to air trapping, with residual volume (RV) serving as a key indicator of small airway dysfunction [40]. The RV is elevated before spirometric abnormalities in asthma and correlates with small airway inflammation in COPD and peripheral airway resistance in asthma. The RV/total lung capacity (RV/TLC) ratio is a useful marker of air trapping, because TLC often increases in obstructive diseases [40]. This ratio is inversely correlated with FVC and is higher in severe asthma than in non-severe asthma, although the age- and sex-adjusted predicted values provide better accuracy. Airway resistance, measured via plethysmography, detects changes more sensitively than spirometry and is useful for assessing bronchodilation [40]. However, their lack of specificity for small airways limits their application in evaluating distal airway diseases [40].
4. Impulse Oscillometry (IOS): IOS is a lung function test that uses oscillating pressure waves (3–20 Hz) applied during tidal breathing to assess the respiratory system mechanics [40,41]. It measures impedance (Z), comprising resistance (Rrs), and reactance (Xrs), which reflect the airway and lung properties [40,41]. Low-frequency oscillations (e.g., 5 Hz) provide insights into small airways, whereas high frequencies (>15 Hz) provide insights into larger airways. The difference between R5 and R20 can indicate small airway pathology, although the exact transition between the small and large airways remains undefined [40,41]. In airway obstruction, Rrs becomes frequency-dependent, with increased low-frequency resistance identifying asthma and COPD. Reactance measurements such as resonant frequency (Fres) correlate better with disease severity and hyperinflation. IOS metrics such as R5–20 and X5 are linked to dyspnea scores, quality of life, and response to exacerbations [40,41]. Inspiratory–expiratory reactance differences (ΔX5) can differentiate asthma from COPD and detect expiratory flow limitation, contributing to dynamic hyperinflation [41]. IOS is sensitive to changes in bronchodilation in asthma and COPD and has applications in bronchiolitis obliterans and environmental exposure assessments. Its simplicity, effort independence, and suitability for noncooperative individuals make it valuable, although coaching is required to avoid artifacts such as tongue movement [40,41].
5. Chest CT for SAD: Chest CT scan is useful for evaluating structural abnormalities in the lungs, which may contribute to airway dysfunction [42]. High-resolution computed tomography (HRCT) is particularly beneficial for assessing small airway changes as it provides detailed images of the lung parenchyma and airway structures [42]. In individuals with SAD, HRCT can reveal features, such as airway wall thickening, air trapping, and mosaic perfusion patterns, which suggest abnormal ventilation in certain lung regions. These imaging findings help identify the presence of small airway disease and assess its severity, complementing other evaluating methods, such as spirometry and IOS [42]. An expiratory CT scan is specifically used to assess SAD by providing images of the lungs during expiration, which can highlight abnormalities that may not be visible during the inspiratory phase [42]. This technique is valuable for detecting air trapping, a key feature of small airway dysfunction that occurs when air is trapped in the lungs owing to incomplete expiration. This method can reveal mosaic attenuation patterns, where areas of low attenuation (indicating air trapping) are observed alongside regions of normal attenuation. These findings are important for evaluating and assessing the severity of small airway disease [42].

## 6. Treatment for OSAS and SAD

### 6.1. Treatment for OSAS

The management of OSAS focuses on alleviating airway obstruction and improving respiratory function during sleep [43]. The first-line therapy is continuous positive airway pressure (CPAP), which delivers a continuous stream of air through a mask to keep the airway open. For more severe cases or individuals with comorbidities, bilevel positive airway pressure therapy (BiPAP) may be used, offering different pressure levels for inhalation and exhalation [43]. Surgical options such as uvulopalatopharyngoplasty or maxillomandibular advancement surgery are considered for individuals with anatomical obstructions. Lifestyle changes, including weight loss, the avoidance of alcohol and sedatives, and sleep hygiene can help reduce symptoms. Oral appliances that reposition the jaw to maintain airway opening are most effective for mild-to-moderate OSAS [43].

### 6.2. Treatment for SAD

Treatment for SAD aims to improve airflow and reduce inflammation in the smaller airways [44]. Bronchodilators, including short-acting beta-agonists (SABAs) and long-acting beta-agonists (LABAs), help relax the smooth muscles of the airways and enhance airflow. Inhaled corticosteroids (ICSs) are anti-inflammatory drugs that reduce airway inflammation and are commonly used in individuals with asthma and COPD. Leukotriene modifiers, such as montelukast, help control inflammation, particularly in asthma [44]. Phosphodiesterase-4 inhibitors play a significant role in suppressing inflammatory cell function, providing anti-inflammatory benefits in respiratory diseases such as chronic COPD and asthma [45]. Roflumilast, a systemically delivered PDE4 inhibitor, has been approved for treating individuals with severe COPD, chronic bronchitis, and a history of exacerbations [45]. Studies that specifically address the use of CPAP or BiPAP in individuals with SAD are lacking. However, extensive research has established the effectiveness of noninvasive positive pressure ventilation (NIPPV) in treating respiratory conditions such as asthma and COPD [46], highlighting its potential therapeutic value in managing SAD and improving respiratory mechanics.

### 6.3. Treatment for OSAS and SAD

When OSAS and SAD coexist, a combined approach is essential to address both conditions simultaneously. CPAP or BiPAP therapy, primarily used for OSAS, can also indirectly benefit SAD by improving airflow and reducing airway collapse during sleep, particularly in individuals with both OSAS and other conditions such as COPD or asthma [43,46]. Inhaled drugs such as ICS, LABA, or LAMA are not standard treatments for OSAS. Therefore, assessing SAD in individuals with OSAS is crucial to ensure the appropriate diagnosis and prescription of targeted medications for managing SAD. A previous study showed that albuterol premedication significantly reduced the risk of OSA in children, particularly in those with moderate to severe OSAS [47].

A multidisciplinary approach involving pulmonologists, sleep specialists, and other healthcare providers ensures better management and tailored therapy, with lifestyle modifications such as weight loss and smoking cessation playing a crucial role. In cases where OSAS is caused by anatomical obstructions, surgical interventions to address these issues can improve airflow in individuals with SAD. Combining therapies while closely monitoring patient responses is the key to improving both sleep quality and overall respiratory function.

## 7. Comorbidities of OSAS and SAD

### 7.1. Comorbidities of OSAS

OSAS is frequently associated with pulmonary, metabolic, cardiovascular, renal, and neuropsychiatric comorbidities [48]. As previously mentioned, the prevalence of OSAS is known to be higher in individuals with asthma and COPD [12,13]. Other pulmonary diseases, such as interstitial lung disease (ILD), are also highly prevalent in OSAS [49]. Among individuals with ILD, 61% had OSA, with 32% classified as mild (AHI 5–15/h), 17% as moderate (AHI 15–30/h), and 9% as severe (AHI ≥ 30/h) [49]. Metabolic syndrome, including hypertension, insulin resistance, hyperlipidemia, and central obesity, is also closely linked to OSAS [48]. Growing evidence suggests that OSAS is an independent risk factor for type 2 diabetes and insulin resistance. One meta-analysis, which included a total of 64,101 participants, shows that OSAS was associated with the incidence of diabetes with the pooled relative risk of 1.62 (95% confidence interval [CI], 1.45–1.80) [50]. Mechanisms include intermittent hypoxia and sleep fragmentation, leading to sympathetic activation and inflammation [50]. Despite shared risk factors, large-scale studies indicate that OSAS independently increases the risk of coronary heart disease, congestive heart failure, and cardiovascular mortality [48]. One previous study revealed that individuals with OSAS had greater odds of developing atrial fibrillation (adjusted odds ratio [OR] = 4.02; 95% CI: 1.03–15.74), coronary heart disease (OR = 4.02; 95% CI: 1.03–15.74), and tachycardia (OR = 3.40; 95% CI: 1.03–11.20) compared to those without sleep apnea [51]. Individuals with OSAS have also been reported to be at high risk for chronic kidney disease (CKD) [52]. One recent study showed that approximately 30% of individuals with an AHI > 15/h also had CKD [52]. Compared to those with no or mild OSAS, the OR for CKD was 2.63 (95% CI: 1.79–3.85) for moderate OSAS and 2.96 (95% CI: 2.04–4.30) for severe OSAS [52]. OSAS is a significant risk factor for stroke, with an estimated two-fold increase in stroke incidence [53]. In a previous cohort study, individuals with an AHI greater than 20/h showed a 4.3 times higher risk for stroke [54]. The presence of dysautonomia in OSAS, along with the circadian activation and fluctuations of the renin–angiotensin–aldosterone system, accelerated atherosclerosis, cardiac arrhythmias, platelet aggregation, hypercoagulability, and endothelial damage, are all reported to be associated with an increased risk of stroke in OSAS [48]. OSAS is associated with an increased incidence of GERD, with a higher OR of 1.19 (95% CI: 1.11–1.28) [55].

### 7.2. Comorbidities of SAD

The factors, including smoking, air pollution, and occupational hazards, contribute to inflammation and damage to small airways, thereby exacerbating airway remodeling [15]. Chronic respiratory diseases are common comorbidities of SAD [30,31]. In COPD individuals, approximately 50% have SAD [30]. The prevalence of SAD in asthma ranges from 53% to 90%, depending on the severity of asthma [31]. SAD was identified in 47.5% of individuals with connective tissue disease-associated ILD and was associated with lower vital capacity and diffusion capacity compared to those without SAD [56]. Individuals with GERD were reported to have a higher prevalence of SAD, at 34%, even in the absence of respiratory symptoms [57]. SAD was reported in 47% of individuals with heart failure and was associated with reduced exercise capacity and lower survival rates, especially in those with a left ventricular ejection fraction of less than 40% [58].

### 7.3. Comorbidities of OSAS and SAD

Both SAD and OSAS are associated with a higher prevalence of pulmonary diseases, including COPD [13,30], asthma [12,31], and ILD [49,56], as well as GERD [55,58] and cardiovascular involvement [51]. The overlap in comorbidities highlights potential interconnected pathophysiological mechanisms. Systemic inflammation and oxidative stress are key shared factors that may contribute to disease progression in both OSAS and SAD [24,25].

## 8. Prognosis of OSAS and SAD

### 8.1. Prognosis of OSAS

In a 5-year follow-up study, the primary event rate, including death, cardiovascular events, stroke, and heart failure with hospitalization, was 7% in individuals with mild-to-moderate OSAS (AHI 5–30/h), 10% in individuals with severe OSAS (AHI 30–55/h), and significantly higher at 33% in individuals with very severe OSAS (AHI > 55/h) [59]. The study also found that, while CPAP effectively reduced cardiovascular risk in severe OSAS, it was insufficient for very severe OSAS, suggesting the need for additional systemic medical treatment in these individuals [59]. In a 15-year study, long-term CPAP users in OSAS (>5 years) were found to be 5.63 times more likely to be alive at the study’s end compared to non-CPAP users (95% CI: 4.83–6.58) and 1.74 times more likely than short-term CPAP users (≤5 years) (95% CI: 1.49–2.02) [60]. Respiratory mortality was more common in individuals with OSAS, particularly among those who did not use CPAP [60].

### 8.2. Prognosis of SAD

The prognosis of SAD has been relatively limited in research. However, in a study with a median follow-up of 12.8 years, individuals with SAD were found to have an increased risk of all-cause mortality (hazard ratio [HR], 1.31; 95% CI, 1.26–1.36), cardiovascular mortality (HR, 1.39; 95% CI, 1.29–1.51), respiratory mortality (HR, 2.20; 95% CI, 1.92–2.51), and neoplasm mortality (HR, 1.23; 95% CI, 1.17–1.29) [61].

### 8.3. Prognosis of OSAS and SAD Overlap

As of now, there are no known studies specifically addressing the prognosis of the overlap between OSAS and SAD. However, a study surveying OSAS combined with asthma or COPD revealed the 10-year mortality rates for non-overlapping conditions: asthma, OSAS, and COPD were 54.2%, 60.4%, and 63.0%, respectively [62]. The overlap syndromes had the following mortality rates: COPD-OSAS (53.2%), asthma–COPD (62.1%), asthma-OSAS (63.5%), and the triple overlap of asthma–COPD-OSA (67.8%). Additionally, individuals with OSAS who were non-adherent to CPAP therapy had an adjusted risk of death 1.78 (95% CI: 1.13–2.82) times higher compared to those who used CPAP [62].

## 9. Future Directions

### 9.1. Potential for Personalized Treatment Strategies for Individuals with OSAS and SAD

As our understanding of both OSAS and SAD advances, there is growing potential for developing personalized treatment strategies tailored to the unique needs of individual individuals. Table 1 lists the characteristics of the SAD and OSAS groups. Personalized medicine based on phenotypic and environmental factors can help optimize therapy for individuals with both conditions. For OSAS, advancements in the identification of specific subtypes based on anatomical features or disease severity could lead to more targeted interventions, such as customized positive airway pressure devices or surgical approaches. Similarly, for SAD, personalized treatment regimens may include specific combinations of bronchodilators, anti-inflammatory agents, and lifestyle interventions, considering the patient’s underlying respiratory diseases and the severity of small airway involvement. The integration of genomic data, biomarkers, and more precise imaging techniques could further enhance the ability to predict responses to therapies and improve long-term outcomes.

### 9.2. Gaps in Current Knowledge and Research Needs

Despite significant progress in the understanding of OSAS and SAD, critical gaps remain in the current knowledge and substantial research needs. One key area requiring further investigation is the precise relationship between OSAS and SAD, particularly how these conditions interact at the molecular and pathophysiological levels. More research is needed to explore the impact of OSAS treatment (e.g., CPAP or BiPAP) on small airway function and vice versa. Additionally, the long-term effectiveness and safety of combined therapies in individuals with both OSAS and SAD requires further study. There is also a lack of reliable biomarkers for the early detection and monitoring of SAD, which limits our ability to intervene earlier in the disease process. Furthermore, research should focus on optimizing diagnostic tools and imaging techniques for the assessment of both conditions as well as exploring novel therapeutic agents and strategies. Finally, randomized controlled trials are required to establish evidence-based guidelines for the management of individuals with concurrent OSAS and SAD, particularly in diverse patient populations. Addressing these gaps is crucial for improving patient outcomes and refining therapeutic approaches.

## 10. Conclusions

The interplay between OSAS and SAD is a complex but critical area of study with significant implications for respiratory health. Both conditions share common risk factors, including obesity and age, and their coexistence can exacerbate respiratory symptoms and increase the risk of cardiovascular and metabolic complications. An enhanced understanding of the shared pathophysiological mechanisms, including airway inflammation, oxidative stress, and structural airway changes, is essential for developing integrated management strategies. Future research should focus on elucidating the molecular links between OSAS and SAD, optimizing diagnostic approaches, and personalizing treatment strategies to improve outcomes in affected individuals. Addressing the current knowledge gaps is vital for improving care and ensuring that individuals with overlapping respiratory disorders receive comprehensive and effective treatment.

## Figures and Tables

**Table 1:** Characteristics of small airway disease and obstructive sleep apnea syndrome.

	Small Airway Disease	Obstructive Sleep Apnea Syndrome
Definition	A spectrum of conditions affecting the airways < 2 mm in diameter, characterized by inflammation and obstruction [2]	A sleep disorder characterized by intermittent upper airway obstruction during sleep [1]
Pathophysiology	Inflammation, obstruction, and remodeling of the small airways [2,23]	Intermittent upper airway obstruction caused by anatomical factors and reduced muscle tone during sleep [1,20]
Interrelationship	Inflammatory mediators released during apneic episodes of OSAS cause airway inflammation and remodeling [24]. Hypoxia–reoxygenation cycles: Repeated cycles of OSAS induce inflammation in the large and small airways [24,25]. Oxidative Stress: Apnea and hypopnea episodes lead to oxidative stress that impairs mucociliary clearance, promoting mucus hypersecretion [25]. Negative intrathoracic pressure: During apneas, increased negative pressure causes dynamic airway collapse and thoracic fluid accumulation [27]. Obesity and airway narrowing: Fat deposition around large and small airways narrows the airways, increasing obstruction risk [9,19].	
Prevalence	Prevalence varies due to differing diagnostic criteria [14] Association with smoking, environmental pollutants, occupational exposures, and obesity [14] 43.5% prevalence in Chinese population [14] Ranges from 5% in Tartu, Estonia, to 34% in Mysore, India [15] SAD in COPD: Present in >50% of individuals [16] SAD in asthma: Occurs in 40% to 70% of asthma individuals [17]	Ranges from 9% to 38% in general adult population [8] Prevalence varies due to factors like age, sex, and BMI [9,10,11,12,13] 69% obese individuals have OSA [9] Male-to-female ratio of 3.5:1 [10] 68% asthma have OSAS and increases with asthma severity [12] 50% COPD have OSAS and increases with COPD severity [13]
Symptoms	Cough, wheezing, and shortness of breath, especially during exertion [39]	Breathing pauses during sleep, snoring, excessive daytime sleepiness, and fatigue [32]
Risk Factors	Environmental pollutants [15] Cigarette smoke [15] Occupational exposure [15] Obesity [15] Comorbid conditions: Asthma, COPD, and GERD [15] Genetics: Family history of respiratory diseases [15]	Obesity [28] Older age [28] Sex: Men are at higher risk [28] Lifestyle factors: Smoking and alcohol consumption [28] Nasal congestion [28] Anatomical abnormalities [28] Genetic predisposition [28]
Evaluation	Clinical assessment: To identify symptoms and risk factors [39] Pulmonary function tests: FEF25–75 [39] and FEV3/FVC [40] Plethysmography: Measures air trapping and hyperinflation [40] Impulse oscillometry: Assesses airway resistance and reactance [40,41] CT scan: Expiratory CT detects air trapping and mosaic patterns [42]	Clinical assessment: Sleep patterns, snoring, apneas, daytime sleepiness, risk factors [32] Sleep questionnaires: ESS, PSQI, STOP-Bang [32,33] Polysomnography: Records EEG, EMG, EOG, respiratory effort, oxygen levels, airflow, and AHI [34] Home sleep apnea testing: Portable home monitoring [34] Sleep endoscopy: Visualizes nasal and oropharyngeal abnormalities [35] Cephalometric radiography: X-ray to assess jaw, airway, and skeletal structure [36] Modified Mallampati classification [37] Maxillofacial/neck CT: Imaging to evaluate airway and soft tissue [36] MRI: High-resolution imaging of soft tissues and brain [36] Ultrasound: Non-invasive imaging of airway structures [38]
Treatment Options	Bronchodilators: SABA, LABA, or LAMA [44] Inhaled corticosteroids (ICSs): Reduce airway inflammation [44] Leukotriene modifiers: Control inflammation [44] PDE4 inhibitors: Control inflammation [45] NIPPV: Beneficial for severe asthma, COPD, and potentially SAD [43]	CPAP to keep airways open [43] BiPAP for more severe cases or with comorbidities [43] Surgical options: UPPP or maxillomandibular advancement for anatomical obstructions [43] Lifestyle changes: Weight loss, avoiding alcohol and sedatives, and sleeping on one’s side [43] Oral appliances: Jaw repositioning [43]
Comorbidities	In total, 50% COPD have SAD [30] In total, 53–90% asthma have SAD, depending on the severity of asthma [31] In total, 47.5% ILD have SAD [56] In total, 34% GERD have SAD [57] In total, 47% heart failure have SAD [58]	In total, 68% asthma have OSAS, prevalence increases with asthma severity [12] In total, 50% of COPD have OSAS, prevalence increases with COPD severity [13] In total, 61% ILD have OSAS [49] OSAS associated with an OR of 1.62 for the incidence of diabetes [50] OSAS associated with an OR 4.02 for both atrial fibrillation and coronary heart disease [51] In total, 30% of individuals with AHI > 15 had CKD [52] OSAS with AHI > 20/h had a 4.3 times higher risk for stroke [54] OSAS have OR 1.19 for GERD [55]
Prognosis	Associated with increased all-cause (HR 1.31), cardiovascular (HR 1.39), respiratory (HR 2.20), and neoplasm (HR 1.23) mortality over 12 years [61]	Cardiovascular event rates over five years were 7% in mild-to-moderate OSAS (AHI 5–30/h), 10% in severe OSAS (AHI 30–55/h), and 33% in very severe OSAS (AHI > 55/h) [59] Over 15 years, long-term CPAP users had a 5.63-fold higher survival rate than non-users and 1.74-fold higher than short-term users [60]

Abbreviations: SAD, small airway disease; OSAS, obstructive sleep apnea syndrome; BMI, body mass index; COPD, chronic obstructive pulmonary disease; GERD, gastroesophageal reflux disease; FEF25–75, forced expiratory flow 25–75%; FEV, forced expiratory volume; FVC, forced vital capacity; ESS, Epworth Sleepiness Scale; PSQI, Pittsburgh Sleep Quality Index; EEG: electroencephalography; EMG, electromyography; EOG, electrooculography; CT, computed tomography; MRI, magnetic resonance imaging, AHI, apnea–hypopnea index; SABA, short-acting beta-agonist; LABA, long-acting beta-agonist; LAMA, long-acting muscarinic antagonist; ICS, inhaled corticosteroid; PDE4, phosphodiesterase-4; NIPPV, non-invasive positive pressure ventilation; CPAP, continuous positive airway pressure; BiPAP, bilevel positive airway pressure; UPPP, uvulopalatopharyngoplasty; OR, odds ratio; HR, hazard ratio; ILD, interstitial lung disease; and CKD, chronic kidney disease.

## Data Availability

Data sharing is not applicable.

## Abbreviations

The following abbreviations are used in this manuscript: SAD, small airway disease; OSAS, obstructive sleep apnea syndrome; BMI, body mass index; COPD, chronic obstructive pulmonary disease; GERD, gastroesophageal reflux disease; FEF25–75, forced expiratory flow 25–75%; FEV, forced expiratory volume; FVC, forced vital capacity; ESS, Epworth Sleepiness Scale; PSQI, Pittsburgh Sleep Quality Index; EEG, electroencephalography; EMG, electromyography; EOG, electrooculography; CT, computed tomography; MRI, magnetic resonance imaging; AHI, apnea–hypopnea index; SABA, short-acting beta-agonist; LABA, long-acting beta-agonist; LAMA, long-acting muscarinic antagonist; ICS, inhaled corticosteroid; PDE4, phosphodiesterase-4; NIPPV, non-invasive positive pressure ventilation; CPAP, continuous positive airway pressure; BiPAP, bilevel positive airway pressure; UPPP, uvulopalatopharyngoplasty; FHx, family history; TB, tuberculosis; IPF, idiopathic pulmonary fibrosis; HTN, hypertension; CHF, congestive heart failure; CAD, coronary artery disease; DM, diabetes mellitus; MMEF, maximum mid-expiratory flow; MCT, methacholine challenge test; BD, bronchodilator; PC20, provocative concentration of methacholine causing a 20% decline in FEV1; dFVC, change in forced vital capacity; dFEV1, change in forced expiratory volume in one second; and dMMEF, change in maximum mid-expiratory flow.
